# Supplementary figures and images for: Cardiovascular vulnerability predicts hospitalisation in primary care clinically suspected and confirmed COVID-19 patients: A model development and validation study
Source: PLoS One. 2022 Apr 11;17(4):e0266750. doi: 10.1371/journal.pone.0266750 (PMC9000124; doi:10.1371/journal.pone.0266750)

**S3 Fig**


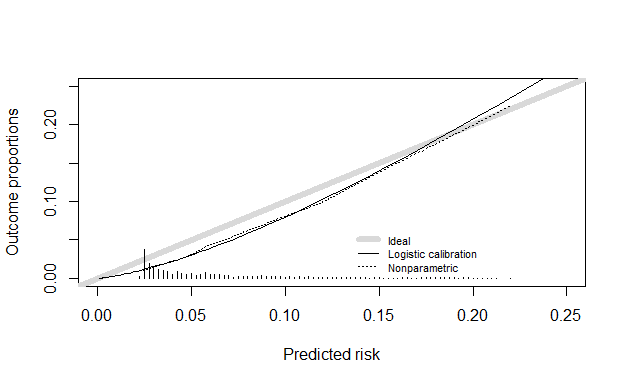


Fig a


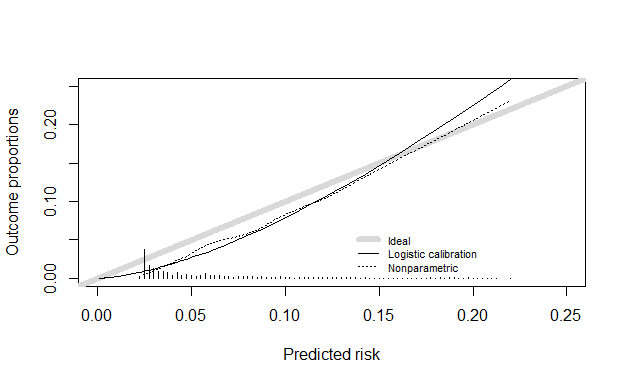


Fig b


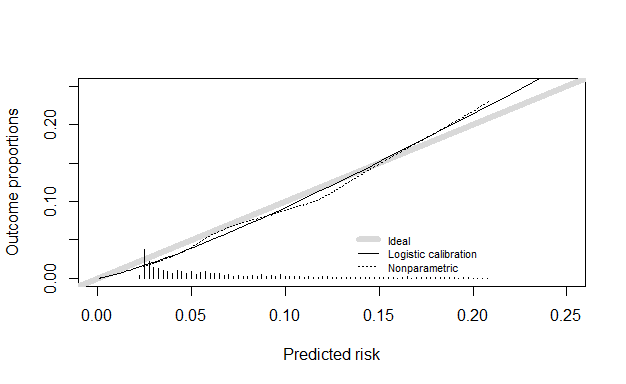


Fig c


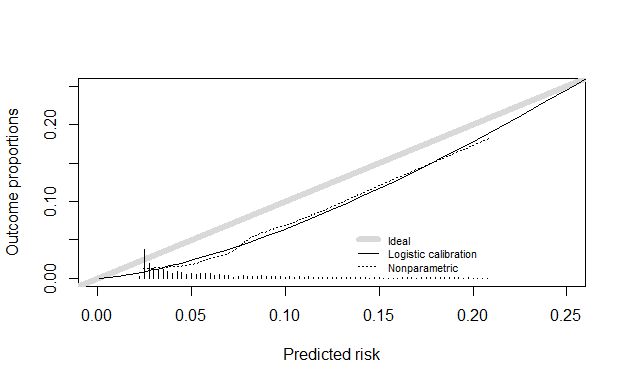


Fig d

Supplement: S1 Fig — Fig a. Calibration plot in the total validation cohort with hospitalisation as the outcome. Fig b. Calibration plot in JGPN validation cohort with hospitalisation as the outcome. Fig c. Calibration plot in AHA validation cohort with hospitalisation as the outcome. Fig d. Calibration plot in ANH validation cohort with hospitalisation as the outcome. (DOCX) [file pone.0266750.s001.docx]
