# Supplementary material for: Cardiovascular vulnerability predicts hospitalisation in primary care clinically suspected and confirmed COVID-19 patients: A model development and validation study
Source: PLoS One. 2022 Apr 11;17(4):e0266750. doi: 10.1371/journal.pone.0266750 (PMC9000124; doi:10.1371/journal.pone.0266750)
Supplement: S1 File — (DOCX) [file pone.0266750.s003.docx]

**S1**

**Methodological details**

Data were collected from three routine primary care databases: the Julius General Practitioner’s Network (JGPN) University Medical Center Utrecht, the Academic Network of General Practice at VU University medical center in Amsterdam (ANH VUmc), and the Academic General Practitioner’s Network at Academic Medical Center Amsterdam (AHA AMC).(12–14) Two databases (JGPN and ANH VUmc) were used to identify patients for the development of the prediction model (i.e. development cohort) and all three databases (JGPN, ANH VUmc and AHA AMC) were used to identify patients for the temporal validation (i.e. the validation cohort).

For the development cohort, data were collected from March 1 2020 to June 1 2020 (the ‘first wave’ of COVID-19 infections in the Netherlands). Data for the validation cohort were collected from September 1 2020 until April 15 2021 from JGPN, and AHA AMC and ANH Vumc patients were included from June 1 2020 until December 31 2020 for validation (the ‘second wave’ of COVID-19 infections).

For identification of the study population and data collection, the same methods were applied in all three databases. Dutch primary care physicians record diagnoses and clinical symptoms in the electronic medical records as diagnostic codes using the International Classification of Primary Care (ICPC) coding system. The primary care physicians participating in the JGPN, AHA AMC and ANH Vumc databases are trained in and experienced with using ICPC codes.

For the development cohort, COVID-19 suspected patients were identified using the ICPC codes R74 (acute upper respiratory infection), R81 (pneumonia) and R83 (other respiratory infection). At the time, primary care physicians were recommended to use R81 and R83 for indicating COVID-19 suspected and COVID-19 confirmed cases, respectively, for lack of COVID-19 specific ICPCs. Records of patients labelled with ICPC R74 (unspecified acute upper respiratory infection) were manually screened for COVID-19 suspicion in the consultation text by three (primary care) clinical scientists (FSvR, LPTJ, and SvD) and cases of doubt were discussed until agreement was reached. Patients with ICPC R74 yet without having a synonym of or reference to COVID-19 suspicion or related symptoms in the consultation text were excluded from the cohorts.

During the ‘second wave’ standardized coding became available in primary care: ICPC R83 and R83.03 to code confirmed COVID-19 patients in the electronic medical record. Thus, for the validation cohort only confirmed cases were included based on ICPCs R83 and R83.03.

Of all included patients, baseline characteristics (i.e. age; sex; relevant diagnoses; and if available Body Mass Index (BMI), oxygen saturation and C-Reactive Protein (CRP)) were collected. (History) of relevant diseases (i.e. cardiovascular disease, type 2 diabetes mellitus, hypercholesterolemia, hypertension, pulmonary diseases, cancer) were identified using ICPC. S2 Table contains all ICPC codes that were used for this study.
